# Injury Prevention Programs in Youth Football: A Narrative Review of the FIFA 11+ and FUNBALL Programs

**DOI:** 10.7759/cureus.82232

**Published:** 2025-04-14

**Authors:** Mohammed A Khan, Saad A Al Sehemi, Omar A Alahmadi

**Affiliations:** 1 Physical Therapy, Physioplans Physical Rehabilitation Centre, Medina, SAU; 2 Physical Therapy, King Salman Medical City, Medina, SAU

**Keywords:** fifa 11+ program, funball program, neuromuscular control, sports injury prevention, sports safety, strength-training, youth

## Abstract

Football (soccer) is a dynamic sport popular among youth, but it poses risks of injuries such as ligament tears, fractures, and concussions. Injury prevention programs are essential for safeguarding young athletes’ health and performance. This review evaluates two key programs: the 11+ and FUNBALL. The 11+ program is a structured warm-up designed for young female players, emphasizing balance, coordination, agility, and neuromuscular control through dynamic stretches, strength drills, and plyometric exercises. It significantly reduces injury rates, particularly severe and overuse injuries. The FUNBALL program targets young male players, focusing on football-specific movements like sprinting, cutting, and ball-handling. Its flexible design incorporates core stability, balance, and plyometric training, effectively reducing thigh injuries and moderate-to-severe injuries. While both programs utilize multi-component strategies to reduce injury incidence and severity, they differ in target populations and delivery methods. The 11+ program integrates into pre-session routines, whereas FUNBALL offers post-warm-up flexibility with progressive levels tailored to players’ development. Tailored, evidence-based programs like these are vital for mitigating injury risks in youth football, enhancing safety, supporting athletic growth, and fostering long-term participation in the sport.

## Introduction and background

Football (soccer) enjoys global popularity among youth [1]. However, the dynamic nature of the sport, characterized by high-speed movements, frequent collisions, and sudden changes in direction, exposes young players to a significant risk of injury [2,3]. Common injuries include sprains, strains, muscle contusions, and more serious conditions such as ligament tears (particularly ACL injuries), fractures, and concussions [3-6]. These injuries can have significant physical and psychological consequences for young athletes, impacting their short-term and long-term athletic development and overall well-being [7-12].

The high incidence of injuries in youth football necessitates the implementation of effective injury prevention strategies. Recognizing this significant injury burden, there has been a growing interest in developing and evaluating injury prevention programs tailored to the specific demands of football. These programs aim to address the underlying factors contributing to injuries by enhancing physical attributes such as strength, flexibility, agility, and neuromuscular control [13-18]. By improving these key components of athletic performance, players can better withstand the demands of the game, reduce their risk of injury, and ultimately enhance their overall performance and enjoyment of the sport [13,19-22].

This narrative review compares and analyzes two prominent evidence-based programs: the 11+ program and the FUNBALL program. The 11+ program, a comprehensive warm-up routine for young female footballers focused on improving balance, coordination, agility, and neuromuscular control [17], was developed to address the specific needs of this demographic. Its implementation was intended to be integrated into regular training and match warm-ups. Simultaneously, the FUNBALL program emerged as another approach, emphasizing dynamic exercises within plyometrics and running/sprinting categories, alongside ball-handling activities to enhance agility and coordination in youth players [18]. This review will delve into the evidence surrounding the efficacy of these two programs in mitigating injury risks within the context of youth football. While the first study on the 11+ program, a cluster randomized controlled trial (RCT) in young female players, was published in 2008 [17], an updated version, the FIFA 11+ Injury Prevention Program, now exists [19]. However, this review focuses on the original 11+ program.

## Review

This narrative review synthesizes the findings from two primary studies that investigated the effectiveness of injury prevention programs in youth football: Soligard et al. [17], which evaluated the 11+ program, and Obertinca et al. [18], which assessed the FUNBALL program. Both studies employed distinct methodologies and targeted different demographics within youth football.

The 11+ program

The study by Soligard et al. [17] focused on young female footballers aged 13-17 years in Norway. This cluster RCT involved 125 football clubs divided into intervention and control groups across an eight-month league season. The intervention group, comprising 1055 players from 52 clubs, implemented the 11+ program as a comprehensive warm-up routine integrated into every training session and match, with a reported adherence rate of 77%. The control group consisted of 837 players from 41 clubs. Over the course of the season, the intervention group accumulated 49,899 exposure hours (16,057 in matches and 33,842 in training), while the control group accumulated 45,428 hours (14,342 in matches and 31,086 in training).

The primary outcome of the study was injury incidence. A total of 376 injuries were recorded, sustained by 301 players (16% of participants). Within this, 161 injuries were recorded in the intervention group and 215 in the control group. The results demonstrated a statistically significant reduction in overall injury risk rate ratio (RR) 0.68 (95% CI 0.48 to 0.98). The study concluded that the 11+ program was effective in reducing the incidence of injuries in young female footballers.

The FUNBALL program

The study by Obertinca et al. [18] investigated the impact of the FUNBALL program on young male footballers in Kosovo. The study involved 1027 players from 21 football clubs and 70 teams, with an average age of 15.2 ± 1.6 years and 5.0 ± 1.8 years of football experience. The intervention group consisted of 524 players, while the control group comprised 503 players. The FUNBALL program was implemented after the initial warm-up, at least twice weekly, with an average adherence rate of 72.2% during the study period, which lasted from August 2021 to May 2022. The intervention group accumulated 53,454 exposure hours (44,437 training and 9,017 match hours), and the control group reported 52,938 hours (44,272 training and 8,666 match hours).

The primary outcome measure was the overall injury incidence rate. A total of 319 injuries were recorded, with 132 in the intervention group and 187 in the control group. The FUNBALL program demonstrated a statistically significant reduction in the overall injury rate, with an incidence rate ratio (IRR) of 0.69 (95% CI 0.55 to 0.87), representing a 31% reduction. The study also noted a reduction in both match and training injuries. The findings suggested that the FUNBALL program effectively reduces the overall injury burden in young male athletes.

Participant characteristics and intervention details

Table 1 summarizes each study's key attributes, including study design, participant demographics (age & sex), and the duration of the study.

**Table 1:** Characteristics of Studies RCT: Randomized controlled trial

Study	Location	Sex	Age (Years)	Study Design	Program Name	Sample Size	Intervention Group	Control Group	Duration	Reference
FIFA 11+	Norway	F	13-17	Cluster RCT	Warm-up	1892	1055	837	8 Months	Soligard et al. 2008 [17]
FUNBALL	Kosovo	M	13-19	Cluster RCT	FUNBALL	1027	524	503	9 Months	Obertinca et al. 2024 [18]

Table 2 provides a more detailed overview of the participant characteristics, highlighting the differences in gender, age, and levels of participation between the two studies.

**Table 2:** Participants' Characteristics

Study	Age (years)	Sex	Training Experience	Level/Sport	Reference
FIFA11+	15.4 ± 0.7	Female	-	Youth football	Soligard et al. 2008 [17]
FUNBALL	15.2 ± 1.6	Male	5.0 ± 1.8 Years	Regional and Super League (Under 15, Under 17, Under 19 age groups)	Obertinca et al. 2024 [18]

The 11+ program was described as a comprehensive warm-up routine integrated into every training and match, focusing on improving awareness and neuromuscular control through dynamic stretches, strength exercises, jumping drills, and neuromuscular activities (Table 3). The program took approximately 20 minutes to complete and was delivered by a trained coach.

**Table 3:** Intervention Details

Program	Components	Frequency	Duration	Delivery Methods	Reference
The 11+	Running exercises, Strength, plyometrics, balance, and Running Exercises	Performed before every training session and match (8-month season).	20 Minutes	Integrated into the warm-up phase; structured routines with progression based on adherence.	Soligard et al. 2008 [17]
FUNBALL	Balance, core stability, hamstring eccentrics, gluteal activation, plyometrics, running/sprinting, and an optional “GAME” category.	Performed at least twice per week during training sessions.	15-20 Minutes	Implemented post-warm-up; progressive levels to ensure proper technique; and non-mandatory engagement element.	Obertinca et al. 2024 [18]

The FUNBALL program was a multi-component intervention performed after the initial warm-up, including exercises targeting balance, core stability, hamstring eccentrics, gluteal activation, plyometric strength, and running/sprinting (Table 3). It was structured into progressive levels and lasted approximately 15-20 minutes per session, implemented at least twice weekly. Both programs were coach-led, and adherence was a key factor in their implementation.

Injury incidence, common types, and injury severity

Table 4 presents the injury incidence rates, types of injuries, and injury severity for both the 11+ and FUNBALL programs, highlighting their impact across the respective athlete demographics.

**Table 4:** Comparison of Outcomes Minimal injuries: absence from match and training for 1-3 days; Mild injuries: absence from match and training for 4-7 days; Moderate injuries: absence from match and training for 8-28 days; Severe injuries: absence from match and training for more than 28 days.

Program	Injury Measure	Intervention Group No of Injuries	Control Group No of Injuries	Reference
The 11+	Total injuries	161 (100)	215 (100)	Soligard et al. 2008 [17]
	Injury incidence rate (per 1000 hours)	3.23	4.73	
	Most common injury site	35 (21.7) Knee injuries	58 (27.0) Knee injuries	
	Minimal injuries	27 (16.7)	32 (14.8)	
	Mild injuries	24 (14.9)	34 (15.8)	
	Moderate injuries	63 (39.1)	70 (32.5)	
	Severe injuries	47 (29.1)	79 (36.7)	
FUNBALL	Total injuries	132 (100)	187 (100)	Obertinca et al. 2024 [18]
	Injury incidence rate (per 1000 hours)	2.99	2.99	
	Most common injury site	31 (23.5) Thigh injuries	49 (26.2) Thigh injuries	
	Minimal injuries	18 (13.6)	22 (11.8)	
	Mild injuries	56 (42.4)	70 (37.4)	
	Moderate injuries	41 (31.1)	62 (33.2)	
	Severe injuries	17 (12.9)	33 (17.6)	

The 11+ program reported an injury incidence of 3.23 injuries per 1000 hours in the intervention group compared to 4.73 in the control group, a 35% reduction. Knee injuries were the most common. The program also reduced the proportion of severe and moderate injuries and the average days lost to injury.

The FUNBALL program demonstrated an injury incidence rate of 2.99 injuries per 1000 hours for both the intervention and control groups. The intervention group experienced a 31% reduction in total injuries compared to the control group. Thigh injuries were the most frequent injury site. The FUNBALL program resulted in a lower proportion of severe injuries in the intervention group compared to the control group. The program demonstrated effectiveness in reducing injury severity, as defined by absence from match and training for more than 28 days.

Comparative analysis

Both the 11+ and FUNBALL programs represent structured, multi-component approaches designed to reduce football-related injuries in youth players. While both programs prioritize exercises that enhance strength, balance, coordination, and neuromuscular control, they differ in their specific implementation, target populations, and the emphasis placed on certain aspects of injury prevention.

As highlighted in Table 5, the 11+ program primarily targets young female football players and is implemented before every training session and match. Its focus is on a comprehensive warm-up routine that includes a variety of exercises aimed at improving overall physical preparedness. The FUNBALL program, on the other hand, is designed for young male football players and is performed after the standard warm-up, at least twice per week. While both programs incorporate similar core elements of injury prevention, the timing and specific exercises differ.

**Table 5:** Comparative Overview of 11+ and FUNBALL Injury Prevention

Category	The 11+ Program [17]	FUNBALL Program [18]
Focus on Injury Prevention	Prioritizes exercises enhancing strength, balance, coordination, and neuromuscular control.	Prioritizes exercises enhancing strength, balance, coordination, and neuromuscular control.
Timing	Performed before every training session and match.	Performed after the usual warm-up, at least twice per week.
Target Population	Young female football players.	Young male football players.
Multi-Component Approach	Incorporates strength training, plyometrics, balance exercises, and dynamic movements.	Incorporates strength training, plyometrics, balance exercises, and dynamic movements.
Running Exercises	Running straight ahead, hip out/in, circling, jumping, quick runs (2 repetitions each).	Diagonal and forward running/sprinting (3 repetitions each).
Balance	Single-leg balance with ball, throwing ball, or testing partner (2 × 30 seconds each).	Single-leg stance, Y-balance (varied repetitions across 6 levels).
Core Stability	Plank variations (static, alternate leg, leg lift) and side planks (static, dynamic, leg lift).	Plank, side plank, and straight arm plank with varied durations/repetitions.
Hamstring Muscles	Nordic hamstring lower with 3 progression levels (3-15 repetitions).	Nordic hamstring and hamstring walk-outs (varied repetitions across 5 levels).
Gluteal Activation	Squats (heels raised, walking lunges, one-leg squats) with progression levels.	Squat lunges, head-shoulder-hip-knee-ankle activation (6 progression levels).
Plyometric	Vertical, lateral, and box jumps (2 × 30 seconds each).	Forward and skater jumps (varied repetitions across 5 levels).
Games	Not included.	Tic-tac-toe, header game, dribbling game (varied repetitions).
Adherence	High adherence critical for effectiveness.	Adherence less emphasized but structured progression aids engagement.
Key Outcomes	Significant reductions in overall injury rates, overuse injuries, and severe injuries.	Reduction in overall injuries, thigh injuries, and severe injuries.

The timing of the interventions reflects different approaches to integration within training schedules. The 11+ program's consistent integration before every session emphasizes a preventative measure as a routine part of the preparation. In contrast, FUNBALL offers more flexibility in its implementation, being performed after the initial warm-up, which could potentially allow for more focused training on specific injury prevention aspects.

The target populations also differ, with the 11+ specifically developed for young female players and FUNBALL for young male players. This suggests a recognition of potential differences in injury profiles and biomechanical considerations between the genders.

The multi-component approach is a shared characteristic, with both programs incorporating strength training, plyometrics, balance exercises, and dynamic movements. However, the specific exercises and their progression levels vary. For instance, the running exercises in the 11+ focus on straight ahead and varied directional running, while FUNBALL includes more diagonal and forward sprinting. Similarly, the balance and core stability exercises have different specific components and progression levels.

The inclusion of games in FUNBALL, such as tic-tac-toe and header games, highlights an attempt to enhance engagement and motivation, which is less explicitly emphasized in the 11+ program. While the 11+ emphasizes that high adherence is critical for effectiveness, FUNBALL acknowledges that adherence is less strictly emphasized, suggesting that the structured progression within the program itself aids in maintaining player engagement.

Finally, the key outcomes of the programs, as summarized in the "Review of Studies" section, reflect their respective focuses. The 11+ program demonstrated significant reductions in overall injury rates, overuse injuries, and severe injuries, with a particular impact on knee injuries. FUNBALL also showed a reduction in overall injuries, with notable benefits in preventing thigh injuries and severe injuries.

In essence, both programs offer valuable strategies for injury prevention in youth football, but they differ in their specific implementation, target populations, and the nuances of their exercise components. The 11+ provides a standardized, pre-activity routine, while FUNBALL offers a more flexible, post-warm-up approach with an emphasis on engaging activities. The choice of program or the adaptation of elements from both may depend on the specific needs and characteristics of the youth football teams and players.

Discussion 

This narrative review examines two prominent injury prevention programs, the 11+ by Soligard et al. [17] and the FUNBALL program by Obertinca et al. [18], both proven effective in reducing injuries in youth football. By synthesizing findings from RCTs, this review explores their injury prevention mechanisms and effectiveness for male and female players [17,18]. Both programs significantly reduce injury rates, with the 11+ being particularly effective in preventing knee injuries in young females, and FUNBALL focusing on reducing thigh injuries in young males [17,18], while also contributing to a lower overall injury incidence [17,18]. However, it is crucial to consider these findings within the broader context of youth sports injury prevention research. The 11+ program has demonstrated strong evidence of injury reduction, particularly in preventing knee injuries, with Soligard et al. reporting a significant decrease in overall, overuse, and severe injuries among young female footballers [17], aligning with meta-analyses confirming its effectiveness in reducing lower extremity injuries across different age groups and playing levels [20], underscoring the importance of such programs in soccer-related injuries. Furthermore, Owoeye et al. found the 11+ reduced lower extremity injuries in male youth players [21], and review by Althomali et al. highlighted its broad effectiveness, emphasizing the crucial role of adherence [22], supporting findings by Soligard et al. [17]. Conversely, the study by Slauterbeck et al. showed no significant reduction in high school athletes [23], highlighting challenges in real-world translation and the need for integration into coach education [24,25] to maximize impact. The FUNBALL program [18], focusing on dynamic, football-specific exercises, demonstrated a 31% reduction in overall injury incidence, particularly severe and thigh injuries, and showed potential cognitive benefits [26]. Ayala et al. [27] supported multi-component programs like the 11+ and FUNBALL, targeting strength, flexibility, balance, and neuromuscular control. Findings by Crossley et al. [28] in woman's football further reinforce the relevance of multi-component programs like the 11+ for young female players vulnerable to ACL injuries, with similar outcomes seen in handball and basketball [29,30]. 

While the studies by Soligard et al. [17] and Obertinca et al. [18] demonstrate the effectiveness of the 11+ and FUNBALL programs in reducing injury rates, understanding the underlying mechanisms is crucial for optimizing implementation and guiding future program development. Both programs incorporate multi-component exercises targeting key physical attributes known to influence injury risk in football. For instance, the 11+ program, with its emphasis on balance exercises (e.g., single-leg stands, perturbation training), likely contributes to improved neuromuscular control and proprioception, enhancing the body's ability to maintain stability during dynamic movements and respond effectively to external forces, thereby reducing the risk of ankle sprains and knee injuries. The inclusion of strength exercises, particularly those targeting core and lower extremity muscles (e.g., Nordic hamstring exercise), can lead to increased muscle strength and power, providing better support to joints and improving the capacity to absorb impact forces during activities like landing and cutting, potentially mitigating ligamentous and muscle strain injuries. Plyometric exercises in the 11+ further contribute to reactive strength and agility, preparing players for the rapid changes in direction inherent in football. 

Similarly, the FUNBALL program, with its focus on football-specific movements, likely elicits comparable neuromuscular adaptations relevant to the demands of the sport. The inclusion of exercises targeting core stability enhances the control of trunk movements, which is fundamental for efficient force transfer and injury prevention in the lower limbs. Hamstring eccentric exercises, a key component of FUNBALL, are known to improve hamstring strength and the hamstring-to-quadriceps strength ratio, a crucial factor in reducing hamstring strain injuries, which are prevalent in football. The plyometric and agility drills in FUNBALL aim to improve explosive power and the ability to perform rapid and controlled changes in direction, potentially reducing the risk of non-contact injuries associated with these movements. Furthermore, the integration of ball-handling may enhance sport-specific coordination and proprioception, contributing to more controlled and biomechanically efficient movement patterns.

Another factor to consider when comparing the findings of the studies by Soligard et al. [17] and Obertinca et al. [18] is the heterogeneity in their designs and contexts. The 11+ study was conducted over an eight-month period in young female players in Norway, while the FUNBALL study spanned nine months and involved young male players in Kosovo, including those in super and regional leagues. These differences in the length of the observation period, the level of competition, and the geographical location could introduce variability in exposure to injury risk and the types of injuries observed. For instance, higher competition levels might be associated with a greater intensity of play and potentially different injury mechanisms. Similarly, variations in climate and playing surfaces between Norway and Kosovo could also influence injury rates. Therefore, while our review highlights the effectiveness of both programs within their respective study contexts, these methodological and contextual differences should be acknowledged when making cross-study comparisons and considering the generalizability of the findings to other youth football populations.

This narrative review, specifically focusing on youth football, provides valuable insights into the effectiveness of the 11+ and FUNBALL programs, supported by RCTs, highlighting their comprehensive, multi-component approach, reducing specific and overall injury rates, though limited by its narrative format and potential generalizability. The findings from Soligard et al. [17] and Obertinca et al. [18] demonstrate single-season effectiveness, but a key area for future research is to investigate the persistence of these beneficial effects beyond this timeframe, exploring whether neuromuscular and biomechanical adaptations are maintained long-term without continued adherence and the potential need for 'booster' sessions. Longitudinal studies tracking injury rates over multiple seasons are needed to understand the decay rate of protective effects and optimal sustained implementation strategies, considering factors like player motivation and coach adherence. Understanding the mechanisms of injury reduction is crucial; the 11+ likely improves neuromuscular control, proprioception, strength, power, reactive strength, and agility through exercises like single-leg stands and the Nordic hamstring exercise, while FUNBALL likely elicits comparable neuromuscular adaptations relevant to football-specific movements through core stability, hamstring eccentric strength, and plyometrics, potentially also enhancing sport-specific coordination. Future research could benefit from directly investigating the specific neuromuscular and biomechanical adaptations induced by these programs in youth football players. This could involve assessing changes in measures such as muscle strength, balance indices, jump landing mechanics, and kinematic analyses of sport-specific movements following program implementation. A deeper understanding of these mechanisms would not only validate the rationale behind these injury prevention strategies but also inform potential modifications and enhancements to further optimize their effectiveness.

## Conclusions

Structured injury prevention programs, such as the 11+ and FUNBALL, offer compelling evidence for their effectiveness in youth football. The 11+ program has demonstrated significant success in reducing overall injury rates, with a notable impact on knee injuries among young female athletes, as well as a decrease in overuse and severe injuries. Likewise, research on the FUNBALL program highlights its efficacy in lowering the overall injury incidence in young male footballers, particularly in preventing thigh and moderate to severe injuries. These tailored, multi-component interventions, when implemented consistently with active coach involvement, provide a vital means of enhancing player safety, fostering healthy athletic development, and promoting sustained engagement in the sport. The clear benefits underscore the critical importance of widespread adoption and integration of these evidence-based programs within youth football to proactively address and mitigate the inherent risks associated with this popular sport.
